# Formulation and 3D Printing of Collagen/Chitosan Inks: Tailoring the Scaffold Properties

**DOI:** 10.3390/gels12030261

**Published:** 2026-03-21

**Authors:** Teresa Carranza, Mireia Andonegui, Raquel Hernáez, Ana Aiastui, Yi Zhang, Koro de la Caba, Pedro Guerrero

**Affiliations:** 1 BIOMAT Research Group, Escuela de Ingeniería de Gipuzkoa, University of the Basque Country (UPV/EHU), Europa Plaza 1, 20018 Donostia-San Sebastian, Spain; mireia.andonegui@ehu.eus (M.A.); pedromanuel.guerrero@ehu.es (P.G.); 2 Department of Graphic Design and Engineering Projects, Escuela de Ingeniería de Gipuzkoa, University of the Basque Country (UPV/EHU), Europa Plaza 1, 20018 Donostia-San Sebastian, Spain; 3 Multidisciplinary 3D Priting Platform (3DPP), Biogipuzkoa Health Research Institute, 20014 Donostia-San Sebastian, Spain; raquel.hernaezmoya@bio-gipuzkoa.eus (R.H.); ana.aiastuipujana@bio-gipuzkoa.eus (A.A.); 4 ISCIII Platform of Biobanks and Biomodels, Instituto de Salud Carlos III (ISCIII), 28029 Madrid, Spain; 5 ISIS Neutron and Muon Source, Rutherford Appleton Laboratory, Didcot OX11 0QX, UK; 6 BCMaterials—Basque Center for Materials, Applications and Nanostructures, UPV/EHU Science Park, 48940 Leioa, Spain; 7 Proteinmat Materials SL, Avenida de Tolosa 72, 20018 Donostia-San Sebastian, Spain

**Keywords:** collagen, chitosan, 3D printing, extracellular matrix

## Abstract

The development of inks with suitable rheological, physicochemical, mechanical, and biological properties is crucial for the successful fabrication of functional scaffolds via extrusion-based 3D printing. In this study, collagen/chitosan hydrogels with varying polymer ratios were developed and characterized to evaluate their printability and suitability for cartilage tissue engineering. Rheological analyses revealed that all samples exhibited shear-thinning behavior and solid-like viscoelasticity, with the formulation of an 80:20 COL/CHI ratio (20CHI) demonstrating optimal filament formation and dimensional stability. Physicochemical analyses confirmed the preservation of the collagen triple helix and the formation of hydrogen bonding between chitosan and collagen. 20CHI scaffolds showed swelling capacity and high cohesiveness. In vitro studies confirmed the cytocompatibility of the scaffolds with murine fibroblasts and the ability of the scaffolds to promote adhesion, proliferation, and extracellular matrix production of both chondrocytes and adipogenic mesenchymal stem cells (aMSCs). Quantification of sulfated glycosaminoglycan (sGAG) indicated sustained matrix deposition over 28 days, particularly by chondrocytes. These findings demonstrate that 20CHI hydrogel is a promising candidate for 3D printing of biomimetic scaffolds for cartilage regeneration.

## 1. Introduction

The development of hydrogel-based inks for tissue engineering requires a detailed evaluation of their physicochemical, mechanical, and rheological properties to ensure suitability for both 3D-printing and biological applications. Natural polymers are widely used due to their biocompatibility, biodegradability, and similarity to the native extracellular matrix (ECM). Collagen, the most abundant protein in the ECM of articular cartilage, is a natural choice for scaffold biomaterials due to its biocompatibility, biodegradability, and its ability to provide a biomimetic template for cell attachment [1]. Unlike denatured derivatives, such as gelatin, the preservation of the native hierarchical structure of collagen induces mechanical properties that provide structural support and bioactive cues that mimic the native cartilage microenvironment, promoting cell adhesion, proliferation, and ECM synthesis [2]. Additionally, collagen can serve as a reservoir for growth factors and other signaling molecules, further enhancing scaffold bioactivity and promoting tissue regeneration [3]. Nevertheless, the use of natural polymers, such as collagen in 3D-printing inks, presents significant processing challenges because of the risk of collagen denaturation or loss of structural hierarchy during the acidic dissolution and extrusion processes; therefore, other polymers are used to improve the extrudability or shape fidelity of the intended scaffolds [4,5].

Furthermore, the integration of chitosan, a biocompatible polysaccharide derived from chitin, presents opportunities for improving the mechanical and biological properties of collagen-based scaffolds [6]. Chitosan’s unique properties, including its biodegradability, antimicrobial activity, and ability to promote cell proliferation, make it a promising biopolymer for enhancing scaffold performance [7]. Through interactions, specifically through the formation of a physical network stabilized by hydrogen bonding and electrostatic forces between the amino groups of chitosan and the carboxyl groups of collagen, chitosan can reinforce scaffold structure, modulate degradation kinetics, and provide cues for cell behavior, thereby optimizing the regenerative potential of engineered cartilage constructs [8,9].

In this context, 3D printing has emerged as a powerful tool for scaffold fabrication, enabling precise control over scaffold geometry, porosity, and mechanical properties [10]. This technology allows for the creation of complex and patient-specific scaffolds, with tailored architecture to accommodate the unique anatomical features of individual joints [11]. Moreover, 3D printing enables the incorporation of multiple materials and bioactive factors into scaffolds, opening opportunities for enhancing their biological functionality and regenerative potential [12]. In extrusion-based 3D printing, rheological behavior is critical, as it affects shape fidelity and structural integrity. Ideal inks must exhibit shear-thinning behavior, enabling smooth extrusion through fine nozzles, and rapid viscosity recovery post-deposition to maintain the printed shape [13]. Optimizing these properties is essential for ensuring shape fidelity, reproducibility, and cell viability.

While various collagen/chitosan blends have been reported for tissue engineering, this study provides a multi-scale structural analysis, from the molecular level via DSC and FTIR analyses to the supramolecular and fibrillar arrangement using SAXS/WAXS and SANS analyses. This work explicitly links the rheological fingerprints of the inks to the preservation of the collagen triple helix and the accessibility of water within the hybrid matrix, offering a comprehensive understanding of how chitosan modulates collagen assembly for optimal 3D printing and biological performance. In this work, the influence of different contents of chitosan on collagen-based hydrogels was assessed. The printability of the gels was evaluated to determine their suitability for 3D printing, and the physicochemical properties of the resulting scaffolds were analyzed. This comprehensive analysis supports the selection of an optimal blend for 3D printing and further in vitro studies.

## 2. Results and Discussion

### 2.1. Rheological Properties of the Inks

**Figure 1:**
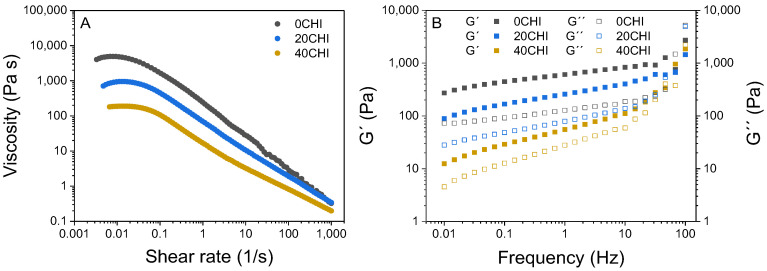
Rheological results for 0CHI, 20CHI, and 40CHI samples: (**A**) shear sweep, and (**B**) frequency sweep.

Steady-state and oscillatory measurements were conducted, as depicted in Figure 1A for the shear sweep and in Figure 1B for the frequency sweep, respectively. All samples exhibited shear-dependent viscosity, which varied with collagen/chitosan ratio (Figure 1A). It was observed that samples with a higher collagen content (0CHI) had higher viscosity, a trend also reported by other authors [14]. Shear-thinning behavior, characterized by a decrease in viscosity with increasing shear rate, was observed for the three samples. The observed shear-thinning behavior (Figure 1A) originates from a complex molecular reorganization within the hybrid network. At low shear rates, the ink maintains high viscosity due to the entanglement of collagen triple helices and chitosan chains, stabilized by a dense network of hydrogen bonds. As the shear rate increases, mechanical stress breaks these physical interactions and forces the biopolymer chains to align in the direction of flow. This behavior is highly desirable for printable solutions, as they are intended to flow easily when subjected to syringe-driven forces [14].

Several rheological models were evaluated to fit the experimental data. The Carreau–Yasuda model was selected as it provided the most accurate fit (R^2^ = 0.99–1.00) and effectively described the Newtonian plateau at low shear rates and the subsequent transition to shear-thinning behavior. As can be seen in Figure 1A and confirmed in Table 1, infinite-viscosity values ($\eta_{\infty }$) were significantly different as a function of the chitosan content used. The higher the chitosan content, the lower the viscosity values at low shear rates. This effect was not noticeable at high shear-rate values. The relaxation time constant (*λ_c_*) was also affected by the chitosan content used, indicating that viscosity started decreasing as a function of the shear rate at slightly higher values.

The rheological parameters derived from the Carreau–Yasuda model provide a clear relationship with the extrusion performance. At high shear rates ($\eta_{\infty }$), low viscosity values ensure that the ink flows easily through the nozzle, facilitating a continuous extrusion process. Conversely, at low shear rates ($\eta_{0}$), high viscosity values provide significant resistance to flow, which is crucial for the material to stop flowing once deposited on the platform. To better understand whether the deposited filament will remain stable and support additional layers without collapsing, it is necessary to analyze the frequency sweeps, which characterize the mechanical integrity of the polymeric system at rest.

Oscillatory measurements (Figure 1B) demonstrated that all the studied samples exhibited solid-like behavior, with the storage modulus (G′) higher than the loss modulus (G″) [15]. The observed solid-like behavior is highly beneficial for the deposition process on the printing platform, ensuring structural stability and allowing the printed layers to support the weight of subsequent layers of material without deformation. The moduli values also depended on the chitosan content, as shown in the shear sweeps, with 40CHI having the smallest difference between G′ and G″ and, thus, the lowest self-supporting capacity, indicating the importance of selecting the collagen/chitosan ratio [16]. These rheological differences among the three samples demonstrate interactions between the two polymers, resulting in changes in their shear and frequency curves, probably due to the penetration of chitosan among collagen fibers, leading to a decompaction of the fibers and altering their flow properties [17]. Additionally, it has been proposed that new interactions may form between both biopolymers through hydrogen bonds between hydroxyl and amino groups of chitosan and the polar side chains of the amino acid residues in collagen [18].

### 2.2. Physicochemical Properties of the Scaffolds

**Figure 2:**
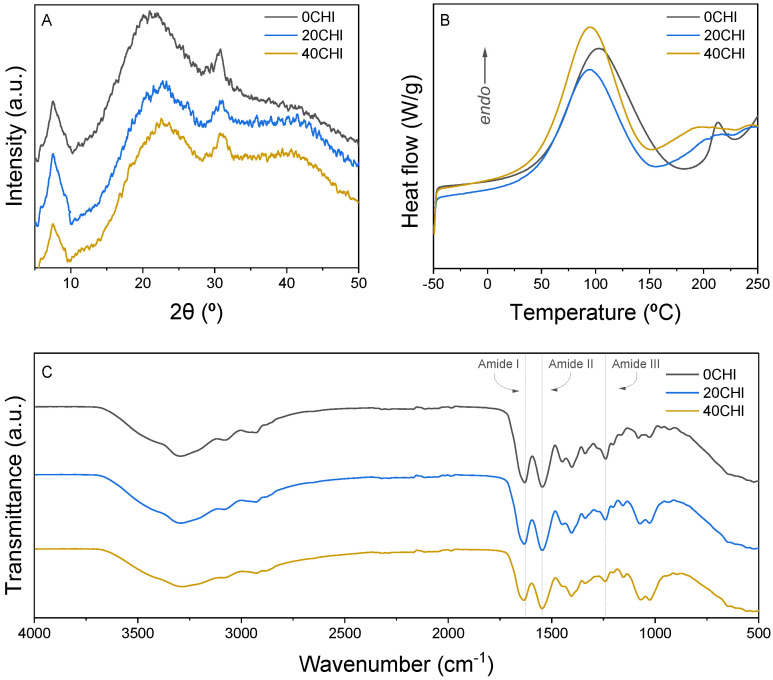
(**A**) XRD spectra, (**B**) DSC curves, and (**C**) FTIR spectra for 0CHI, 20CHI, and 40CHI samples.

The structure of the samples was determined using X-ray diffraction, as can be observed in Figure 2A. The peak around 7.4° represents the distance between the chains in the triple helix of collagen, while a peak around 22° is related to the unordered parts of collagen [19]. The peak around 30° could be related to collagen fibrillar order [20]. Overall, it can be concluded that the structure of the scaffolds is amorphous, with some regions showing more ordered structures.

Regarding thermal properties, DSC curves (Figure 2B) showed two endothermic peaks. The first peak around 100 °C is related to the water release, and the second one around 200–220 °C is attributed to the denaturation of collagen [21]. While this process may overlap with the onset of thermal decomposition, it is well-documented that the major thermal degradation of the collagen backbone occurs at higher temperatures, typically around 300 °C [22,23]. This second transition exhibits a visible broadening and a shift towards lower temperatures in the chitosan-containing formulations compared to the pure collagen control (0CHI). This broadening could reflect a decrease in the cooperativity of the thermal process, indicating that the penetration of chitosan chains into the collagen bundles creates a more disordered molecular arrangement. Furthermore, the shift in the denaturation onset further suggests that the interactions between both biopolymers interfere with the native stabilizing forces of the fibers, effectively reducing the thermal stability of the collagen arrangement [24]. In order to assess the interactions between collagen and chitosan, FTIR spectra are presented in Figure 2C. The broad band from 3600 cm^−1^ to 3000 cm^−1^ is attributed to N-H and O-H vibrations. The amide I band appears at 1629 cm^−1^, 1631 cm^−1^, and 1633 cm^−1^ for 0CHI, 20CHI, and 40CHI, respectively, suggesting the interactions between collagen and chitosan by hydrogen bonding [25]. The amide III band also shifts from 1239 cm^−1^ for 0CHI and 20CHI to 1241 cm^−1^ for 40CHI. These shifts suggest that the amino (–NH_2_) and hydroxyl (–OH) groups of chitosan interact with the carboxyl (–COOH) and amide groups of collagen chains. These physical interactions effectively modulate the molecular environment, reinforcing the hybrid matrix and promoting the rheological and mechanical stability observed in the 20CHI and 40CHI formulations.

**Figure 3:**
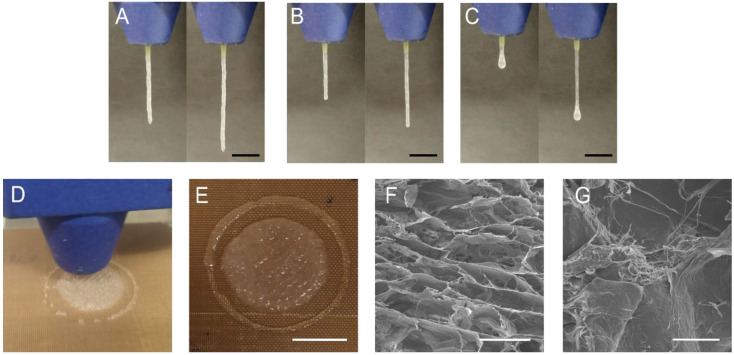
Results of the filament formation test for the (**A**) 0CHI, (**B**) 20CHI, and (**C**) 40CHI inks (scale bar = 10 mm). (**D**) 3D-printing process, (**E**) 20CHI 3D printed scaffold (scale bar = 10 mm), and SEM images of 20CHI scaffold with (**F**) scale bar = 0.5 mm and (**G**) scale bar = 150 µm.

The construction of a precisely defined object through 3D printing requires high control over the filament diameter. This control is conditioned not only by the exit diameter of the needle but also by the viscoelastic properties of the hydrogels [14]. For the 0CHI formulation (Figure 3A), excessive gelation was observed, resulting in a misaligned and irregular filament. On the contrary, the filament formed by 20CHI ink (Figure 3B) was regular in all the sections and showed a smooth appearance, indicating the ability of this ink for 3D printing. Finally, as expected by the previously shown rheological results, 40CHI exhibited insufficient gelation capacity (Figure 3C), as can be observed by the drop formed at the bottom of the extruded filament [26]. Considering the filament with the best shape fidelity, 20CHI ink was selected for 3D printing (Figure 3D) to obtain a 1 mm height scaffold (Figure 3E). The inner structure of this scaffold was investigated by scanning electron microscopy (SEM), revealing a porous structure (Figure 3F). Importantly, the collagen fibril structure was observed (Figure 3G), indicating that the chitosan addition does not compromise the structure of collagen and preserves the triple helix. These results are consistent with the abovementioned rheological results, which showed higher viscosity for samples with higher collagen content, and also with FTIR results, which suggested new interactions between collagen and chitosan, resulting in an easier flow of the material through the nozzle. Furthermore, the printed material retains its shape, preserving the triple helix structure and forming a porous scaffold as required for tissue engineering [27].

**Figure 4:**
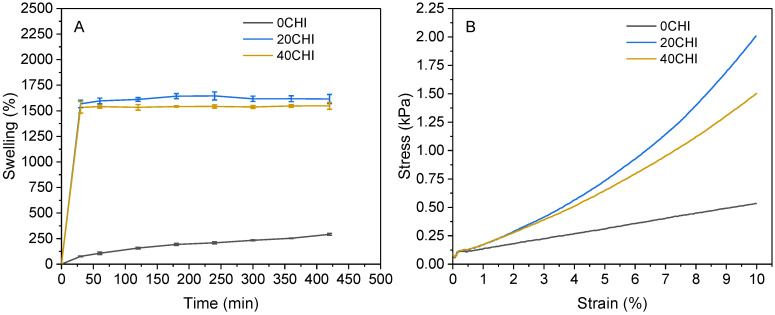
(**A**) Swelling capacity and (**B**) compressive strain-stress curves for 0CHI, 20CHI, and 40CHI samples (n ≥ 4).

In order to assess the behavior of the scaffolds in contact with water, swelling tests were performed, and the results are shown in Figure 4A. 0CHI samples reached a swelling value of 420%, while chitosan-containing samples showed values of 1615 and 1550% for 20CHI and 40CHI, respectively. The difference in the swelling profile is due to the different nature of collagen and chitosan molecules. Collagen is a fibrous protein with a triple helix configuration, and chitosan penetrates between protein chains, loosening the network and increasing the water uptake capacity. It is worth noting that the increase in chitosan content did not increase the swelling capacity of the scaffolds. The stabilization of the swelling capacity in the 40CHI formulation, despite higher chitosan content, can be related to the opposite effects caused by the network loosening and the physical crosslinking when increasing chitosan content. Although the incorporation of chitosan disrupts the tight collagen packing, as evidenced by the DSC and XRD results, the higher content of chitosan in 40CHI increases the number of available functional groups for intermolecular hydrogen bonding. This creates a more densely interconnected hybrid network that, while less stiff than 20CHI due to the lower collagen content, provides enough physical resistance to limit further water uptake. This is consistent with the rheological findings, where 40CHI inks showed the lowest zero-shear viscosity, indicating a different degree of molecular entanglement that ultimately governs the scaffold’s macroscopic behavior. Regarding hydrolytic degradation, the values are shown in Table 2. As can be seen, degradation values ranged around 25% after reaching swelling equilibrium for all the samples, with no statistical differences among them.

In addition to chemical biomimicry, scaffolds must exhibit a mechanical behavior similar to that of the target tissue in order to ensure proper cell differentiation. Therefore, compression tests with 10% deformation were performed, and stress–strain curves are shown in Figure 4B. A 10% compressive strain was selected to ensure a stable mechanical response within the linear viscoelastic region of the scaffolds, accounting for the initial compliance of the hydrogels. This value, although slightly exceeding the physiological range from 2.4% to 8.5% reported by Eckstein and colleagues for patellar cartilage, is representative of the upper limit of physiological deformations and allows for a robust calculation of the compressive modulus [28]. In this work, cohesiveness is defined as the ability of the scaffold to recover its original height and structural integrity after repeated loading. This parameter was calculated as the ratio of the second compressive stress divided by the first one, and, therefore, values close to one indicate a good recovery ability. As can be seen in Figure 4B, all the samples exhibit linear deformation behavior until 2% strain, but the stress values go up sharply in chitosan-containing samples, while collagen samples remain linear. The calculated compressive moduli are shown in Table 2, where the 20CHI samples showed the highest values (12.8 kPa). Although this modulus falls within the physiological range described for native cartilage (10–20 kPa) [29]. This alignment is observed under a specific macroscopic strain of 10 %. It should be noted that, although this deformation allows for a reliable calculation of stiffness within the linear viscoelastic region, the mechanical behavior of native tissue is ultimately determined by its complex anisotropic architecture under dynamic loading, which differs from the static evaluation performed on the scaffolds. Regarding cohesiveness values, it can be observed that chitosan-containing samples demonstrated superior recovery compared to pure collagen after two compression-decompression cycles. The decrease in the compressive modulus for 40CHI is attributed to the steric hindrance caused by the excess of chitosan chains, which disrupts the continuity of the collagen fibrillar network and interferes with the efficient distribution of mechanical stress across the matrix.

Swelling and mechanical results indicated that interactions between chitosan and collagen occurred. It is noteworthy that the total biopolymer concentration remained constant for all samples (3%). The 2.6-fold increase in the compressive modulus for the 20CHI formulation compared to the control (0CHI) demonstrates that the mechanical performance is governed by synergistic intermolecular interactions rather than simple polymer concentration. This reinforcement results from the formation of a hybrid network stabilized by hydrogen bonding between chitosan and collagen, which effectively enhances the stiffness and cohesiveness of the scaffold despite the lower collagen content. Considering the obtained results, the 20CHI formulation, in comparison to 0CHI, was selected to perform advanced characterization by SAXS, WAXS, and SANS, as well as cytocompatibility and neocartilage formation tests.

### 2.3. Advanced Characterization by WAXS, SAXS, and SANS

**Figure 5:**
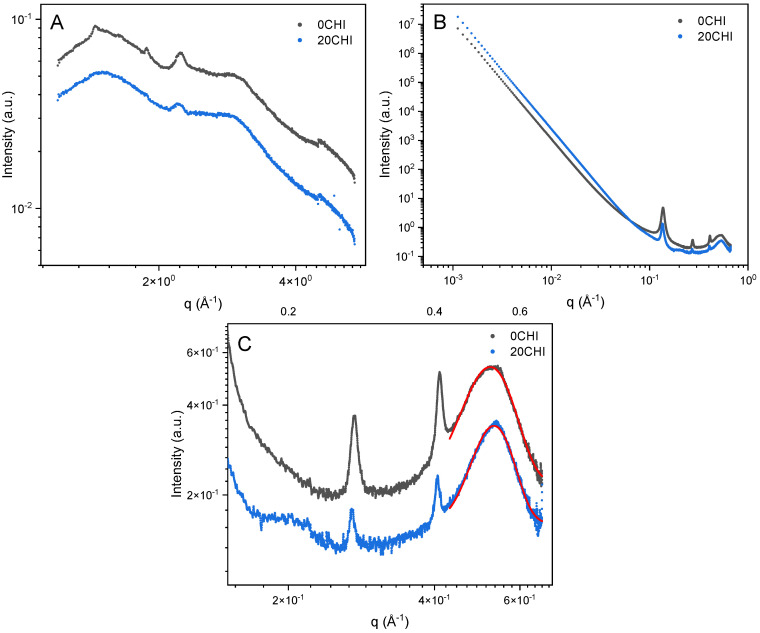
(**A**) WAXS, (**B**) SAXS from 10^−3^ Å^−1^ to 1, and (**C**) SAXS from 3·10^−1^ to 7·10^−1^ Å^−1^ for 0CHI and 20CHI scaffolds. Pink and red lines show the fitting to the Porod’s law.

With a transmitted beam, WAXS analysis revealed the same characteristic triple helical structure of collagen in both 0CHI and 20CHI samples, featuring distinct peaks at q = 2.2 Å^−1^ (Figure 5A). This suggests the molecular integrity of collagen, supporting the observations from XRD, which characterize the surface of the scaffolds. The broad peak at around q = 3.0 Å^−1^ was also observed, which represents the amorphous collagen peptide chains.

SAXS analysis (Figure 5B) showed that the control dry collagen scaffold (0CHI) has smooth interfaces according to Porod’s law, with the scattering intensity featuring a q^−4^ dependence. A similar power law dependence was observed for 20CHI. The sharp peaks between q = 0.10–0.50 Å^−1^ shown in both 0CHI and 20CHI samples indicate a lamellar structure. It is noteworthy that both scattering profiles lacked the set of sharp peaks that originated from the quarter-staggered periodic packing in native collagen fibers. However, a broad peak at q = 0.50–0.60 Å^−1^ (Figure 5C) was observed as a characteristic feature for laterally packed collagen molecules (intermolecular lateral packing, ILP in short), matching a known 1.1–1.2 nm packing distance in dry collagen fibers [20] and the 7.4° peak in XRD results. These observations suggest that collagen was dispersed during processing, then allowed to pack laterally, although randomly positioned axially.

Also, the integrated 1D SAXS profile of the 0CHI sample showed a sharp turn at q = 0.54 Å^−1^, suggesting that this broad feature was significantly oriented at the spots measured, whereas in the 20CHI sample, this is less prominent. It could be related to the better dispersion of collagen assemblies through the interaction with chitosan that moderates the gelation. Additionally, an indicative broad peak was found only in the 20CHI sample at around 0.20 Å^−1^. This may be associated with an intermolecular packing structure of collagen/chitosan in the scaffold, with a d-spacing of 3.1 nm.

**Figure 6:**
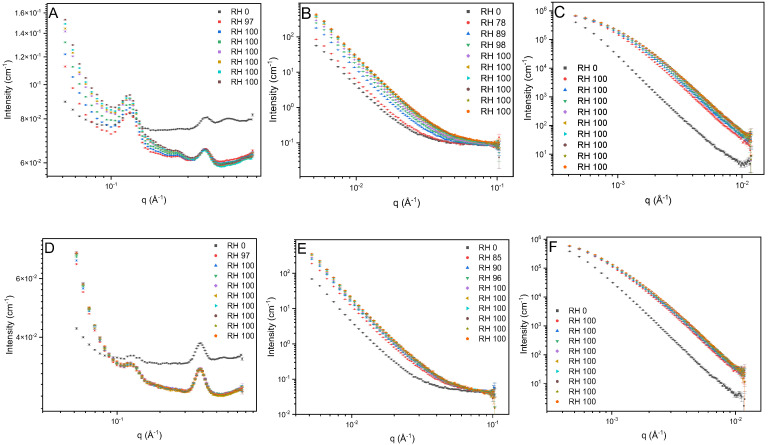
SANS at (**A**,**D**) high q, (**B**,**E**) mid q, and (**C**,**F**) low q, and SANS for 0CHI (**top**) and 20CHI (**bottom**) scaffolds.

The structural features were further studied using humidity-controlled SANS to highlight changes when 0CHI and 20CHI absorb moisture (D_2_O in this case). SANS data of the samples at three q-ranges were plotted separately (Figure 6).

At high q, the peak from laterally packed collagen molecules (ILP peak) and the residual peaks were again observed (Figure 6A,D), matching the SAXS results. At mid q, data were composed of a power law dependence (Figure 6B,E), which continued towards low q before the intensities were affected by multiple scattering (Figure 6C,F). Within the q-range sufficiently separated from the affected part at low q, 0CHI and 20CHI showed power law dependences of q^−3.52^ and q^−3.67^, respectively, suggesting rough interfaces between structural domains across multiple scales. Note that the exponents were generally smaller than those from the SAXS results, due to the different contrast space of SAXS and SANS. SANS is advantageous for resolving the interfaces between polypeptide, polysaccharide, and solvents, as these two biopolymer components have poor contrast under X-ray, as shown in Figure 2A.

Upon changing the RH levels from 2% to over 90% (equilibrium of RH reached within 40 min), D_2_O absorption occurred rapidly in the first 1–2 h, showing an increase in the interface scattering intensity (mid q and low q, the power law component) and the diminished ILP peak and decreased background (high q, constant). If the ILP had remained ordered, D_2_O would have increased the contrast between the triple helical molecules and revealed a stronger peak. Therefore, our observation suggests that the lateral packing of collagen molecules was less ordered upon swelling. The change in background is associated with the hydrogen–deuterium exchange (HDX) on labile sites, which reduces the total incoherent scattering of the sample. The process is much slower in 0CHI control (5 h) compared to 20CHI (1.5 h). This can be an indicator of water accessibility through the scaffold matrix associated with multiple factors, including pore size and connectivity, as well as surface hydrophilicity, confirming the swelling results obtained, where swelling increases significantly with the incorporation of chitosan (Figure 4A).

At mid q and low q, the significant and steady increase in the intensity of the power law component upon exposure to RH = 90% suggests an enhanced contrast associated with the uptake of D_2_O into voids in the matrices. D_2_O in 0CHI slowly dispersed through the matrix over the tested period (16 h), and, in contrast, for 20CHI, intensity increased rapidly for the initial 1 h, followed by negligible differences for the remaining 15 h.

Comparing the humid end products, the power law exponent increased from q^−3.52^ and q^−3.67^ to q^−3.63^ and q^−3.75^ at the hydrated state. The interfaces are slightly smoother when swollen, probably due to the straightened interdomain areas with voids holding up water. It is also noted that, during the tested period, no gel network nor quarter-staggered native collagen was observed. The lack of gel network features suggests that the scaffolds contain mostly bundled integrated collagen molecules rather than unfolded (denatured) gelatin-like chains, indicating that the procedure used in the preparation of the gels does not break down the triple helix of collagen, maintaining its fibrillar structure, and, consequently, the scaffolds maintain their structure in culture media at 37 °C.

### 2.4. Cytocompatibility Assessment and Biochemical Evaluation of Neocartilage Formation

**Figure 7:**
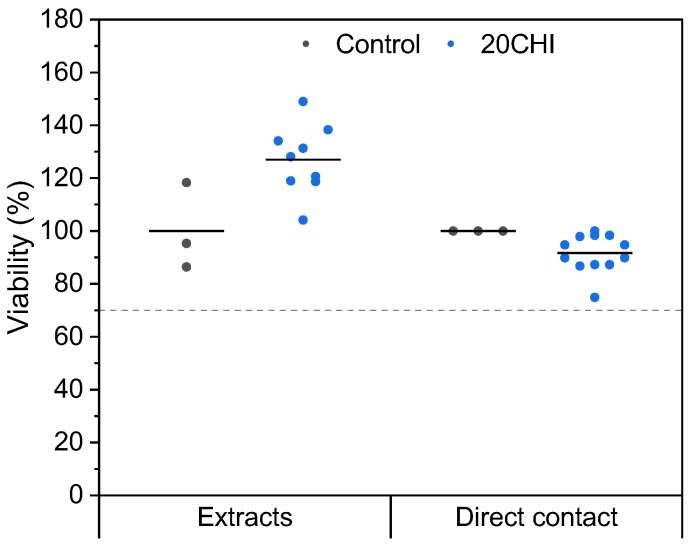
Extract and direct contact cytotoxicity studies following ISO 10993 standard guidelines. The dashed line indicates the minimum viability level required by regulation for a product to be cytocompatible. Solid lines indicate the mean values.

Cytocompatibility is one of the most important features when developing new treatment strategies in tissue engineering. In this study, the scaffold was tested in accordance with the ISO 10993-1 standard for “Biological evaluation of medical devices” [30]. In this case, experiments were performed with 3T3-J2 murine fibroblasts as a cell model. The standard stipulates that cell viability cannot be less than 70%. As can be seen in Figure 7, cell viability is higher than 70% for the extract and for direct contact cytotoxicity studies, indicating the cytocompatibility of the scaffolds.

**Figure 8:**
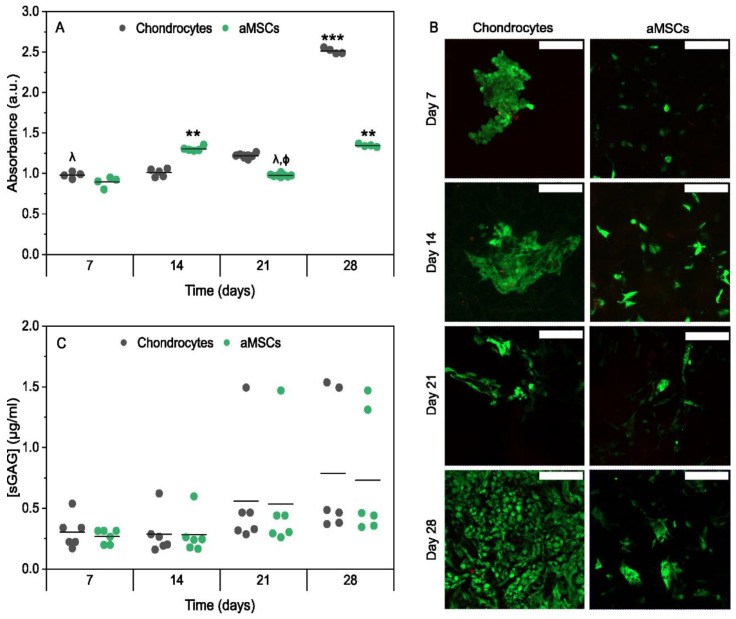
Cell viability and biochemical characterization of CHI20 scaffolds. (**A**) Quantitative long-term viability assay (line represents mean values). (**B**) Fluorescence microscope images showing chondrocytes or aMSCs growing on scaffolds. Live cells are shown in green (calcein-AM), while non-viable cells are shown in red (ethidium homodimer). Scale bar = 200 µm. (**C**) Quantitative assessment of sGAG production by chondrocytes or aMSCs growing on the scaffolds on days 7, 14, 21, and 28 of culture (line represents mean values). λ indicates significant differences (*p* < 0.05) between the viability of chondrocytes on day 28 and cell type and/or culture time. ϕ indicates significant differences (*p* < 0.05) between the viability of aMSCs on day 28 and other cell types and/or culture time. ** *p* < 0.01; *** *p* < 0.001; ϕ *p* < 0.0; λ *p* < 0.05.

Subsequently, to assess whether these scaffolds could support the growth of two cell types, adipose mesenchymal stem cells (aMSCs) and chondrocytes, the proliferation of both cell types was assessed over 7, 14, 21, and 28 days. As can be seen in Figure 8A, both chondrocytes and aMSCs proliferated in culture continuously, with subtle differences over time. For example, chondrocytes showed greater proliferation from day 21 in culture, while aMSCs slowed down and continued to increase in number by day 28. These quantitative results obtained are in agreement with fluorescence microscope images (Figure 8B), confirming high cell viability throughout the extended culture. As illustrated in Figure 8B, aMSCs exhibited slower growth and remained in more isolated clusters at day 28, while the chondrocytes demonstrated a tendency to cover the entire scaffold. Nevertheless, these observations indicate that the scaffolds allow both survival and proliferation of the two types of cells.

In addition, this study aimed to assess the ability of the scaffolds to support extracellular matrix deposition by aMSCs and chondrocytes. Quantification of total sGAG demonstrated increased extracellular matrix (ECM) deposition by days 21 and 28 for both cellular types (Figure 8C). Chondrocytes produced a slightly higher content of sGAG over time. These results indicate that the developed scaffolds effectively support ECM deposition by both aMSCs and chondrocytes. The observed increase in sGAG content at days 21 and 28 suggests active and sustained ECM synthesis, a key indicator of the functional scaffold performance in cartilage tissue engineering. Notably, chondrocytes produced slightly higher levels of sGAG across all time points, consistent with their native chondrogenic phenotype and intrinsic capacity to secrete cartilage-specific ECM components [31]. In contrast, aMSCs exhibited a gradual increase in sGAG production, highlighting the scaffold’s potential to support chondrogenic differentiation in progenitor cells. The comparable ECM deposition by both cell types at later stages suggests that the scaffold microenvironment provides favorable biochemical and biophysical cues for matrix synthesis and cellular function. Although specific chondrogenic markers were not quantified, the sustained sGAG production over 28 days is indicative of the functional activity of the cells and the active secretion of a cartilaginous extracellular matrix. Although steady matrix deposition highlights the potential of the 20CHI scaffold for cartilage regeneration applications, the absence of specific chondrogenic markers and in vivo validation remains a limitation that must be addressed in future studies to definitively confirm its regenerative efficacy.

## 3. Conclusions

The development and characterization of collagen/chitosan-based hydrogels, with tunable rheological and mechanical properties suitable for extrusion-based 3D printing, was carried out in this study. Among the tested formulations, the CHI20 scaffold exhibited optimal shear-thinning behavior, solid-like viscoelastic properties, and appropriate filament formation capacity, enabling the successful fabrication of 3D structures with defined geometry and shape fidelity. Physicochemical analyses confirmed the preservation of the collagen triple helix and the formation of new interactions between collagen and chitosan. The addition of chitosan increased swelling capacity as well as mechanical stiffness and cohesiveness, especially for CHI20 scaffolds, which achieved compressive modulus values within the physiological range of native cartilage. Biological evaluation demonstrated that the CHI20 scaffold was cytocompatible and supported the adhesion, proliferation, and ECM production of both chondrocytes and mesenchymal stem cells. Notably, sustained production of sGAG was observed over 28 days, with chondrocytes showing slightly higher matrix deposition, opening a window for the application of these scaffolds in cartilage regeneration.

## 4. Materials and Methods

### 4.1. Materials

Collagen (COL) was purchased from Proteinmat (Proteinmat, San Sebastián, Spain). Chitosan (CHI) (batch MKBB9037) with a molecular weight of 190 kDa and a degree of deacetylation higher than 75% was supplied by Sigma (Sigma, Barcelona, Spain). NaOH 0.1 M was obtained from Panreac (PanReac, Barcelona, Spain). Dexamethasone, ascorbic acid, Dulbecco’s Modified Eagle’s Medium (DMEM), high-glucose DMEM, L-glutamine, nylon cell strainer from Corning, and CCK-8 kit were purchased from Sigma (Sigma, Barcelona, Spain). Inactive fetal bovine serum (FBS) and penicillin-streptomycin (P/S) were supplied by Lonza (Lonza, O Porriño, Spain). Hank’s balanced salt solution (HBSS), collagenase type II, insulin-transferrin-selenium (ITS), and Fungizone™ were obtained from Gibco (Gibco, Madrid, Spain). TGF-β2 from Peprotech and the Live/Dead kit from Invitrogen were supplied by Thermofisher (Thermofisher Scientific, Madrid, Spain). The Blyscan Kit for the sulfated glycosaminoglycan (sGAG) assay kit was supplied by Biocolor (Biocolor, Belfast, UK). Finally, trypan blue dye was obtained from Sigma (Sigma, Barcelona, Spain).

### 4.2. Preparation of Inks

Collagen gels with different chitosan contents were prepared to obtain scaffolds for 3D printing. Firstly, collagen, chitosan, and 0.5 M acetic acid were mixed using an ultra-turrax T25 (IKA, Staufen, Germany) until homogeneous gels were achieved. The mixing process (2000 rpm, 2 min) was carried out in a cold bath to prevent the dough from heating up. The gels were then loaded into syringes and stored at 4 °C until use. Samples were designated as 0CHI, 20CHI, and 40CHI40 as a function of the COL/CHI ratio, which was 100:0, 80:20, and 60:40, respectively, and the concentration of the biopolymer in solution was 3%.

### 4.3. Rheological Analysis of the Inks

The rheological tests were conducted using the Thermo Scientific Haake RheoStress1 rheometer (IFI, Vigo, Spain). All measurements were carried out with a 35 mm diameter serrated plate-plate configuration and a 1 mm gap between them. Stress sweeps were performed for all samples to determine the linear viscoelastic range (LVR), and a stress value (τ) of 1 Pa was selected for subsequent oscillatory tests. The time-dependent deformation behavior was analyzed by performing frequency sweeps from 0.01 to 100 Hz at a constant temperature. Finally, flow curves were obtained by conducting shear sweeps from 0.001 to 1000 s^−1^. The obtained data were fitted to the Carreau–Yasuda rheological model (Equation (1)) using OriginPro 2021 software (OriginLab Corporation, Madrid, Spain) to estimate zero viscosity (*η*_0_) and infinite viscosity (*η*_∞_), relaxation time constant (*λ_c_*), transition control factor (*a*), and the flow index (*n*).

(1) $$\eta (\dot{\gamma })=\eta_{\infty }+\left(\eta_{0}-\eta_{\infty }\right){\left[1+{\left(\lambda_{c}\dot{\gamma }\right)}^{a}\right]}^{\left(\frac{n-1}{a}\right)}$$

All rheological tests were carried out at a constant temperature of 23 °C and performed in triplicate, with the mean of the measurements reported as the result.

### 4.4. 3D Printing of the Scaffolds

The 3D-printed scaffold consisted of a 14 mm diameter and 2 mm height cylinder, designed using Solid Edge software 2025 (Siemens, Munich, Germany). The structure was sliced with UltiMaker Cura 5.3.1 software (Ultimaker, Utrech, The Netherlands) and printed using a plastic conical nozzle of 14 G (1.55 mm) with the 3D printer domoBIO 2A (Domotek, Hernani, Spain). The printing conditions were set to a layer height of 0.4 mm, a printing speed of 10 mm/s, and an adjustment of the printing flow rate to 200%. Furthermore, an infill density of 100% and a rectilinear pattern at 45°/135° were employed to ensure structural density. The printer’s syringe head configuration was used with both the nozzle and platform temperature set at 23 °C. Scaffolds were freeze-dried for 48 h (Alpha 1-4 LDplus, CHRIST, Osterode am Harz, Germany).

### 4.5. Physicochemical Characterization of the Scaffolds

X-ray diffraction (XRD) measurements were performed by a diffraction unit PANalytical Xpert PRO (PANalytical, Almelo, The Netherlands). The radiation was generated from a Cu-Kα (λ = 1.5418 Å) source operating at 40 kV and 40 mA. The diffraction data were collected from 2θ values from 5° to 50°.

Differential scanning calorimetry (DSC) was performed with a Mettler Toledo DSC 822 (Mettler Toledo, Madrid, Spain). The assays were carried out from −10 °C to 250 °C at 10 °C/min under a nitrogen atmosphere.

An Alpha II plus spectrometer (Bruker, Logroño, Spain) equipped with a ZnSe ATR crystal accessory was used to obtain the Fourier transform infrared (FTIR) spectra. All the samples were analyzed in the spectral range from 4000 to 700 cm^−1^ at 4 cm^−1^ resolution. 32 scans were performed per sample.

Extrusion tests were carried out to ensure adequate extrudability of the inks with the 3D printer head. For this, images of the selected 3D-printing flow rate were taken, and images of the formed filaments were also captured. The images were processed using ImageJ software v1.41 [32].

A Hitachi S-4800 scanning electron microscope (Hitachi, Madrid, Spain) with an acceleration voltage of 15 kV was used. Gold-coated samples were prepared using a JEOL fine-coat ion sputter JFC-1100 (Izasa, Madrid, Spain) under an argon atmosphere.

To perform the swelling test, dried samples were weighed ($W_{0}$) and immersed in PBS (pH 7.4) at 37 °C. The samples were weighed at different time points ($W_{t}$) after being drained with filter paper to remove excess liquid from the surface. The swelling capacity was calculated using Equation (2).

(2) $$Swelling\;(\%)=(W_{t}-W_{0})/W_{0}\cdot 100$$

The degradation occurred after the swelling process was calculated by drying the hydrated samples in the oven at 105 °C for 24 h. The degradation degree was calculated using Equation (3).

(3) $$Degradation\;(\%)=(W_{0}-W_{f})/W_{0}\cdot 100$$

where $W_{0}$ is the initial weight, also calculated for the swelling test, and $W_{f}$ is the final dry sample weight.

Once the scaffolds reached the maximum swelling, they were tested mechanically under 10% compressive deformation using a TA.XT.Plus C Texture Analyzer (Aname Instrumentación Científica, Madrid, Spain). A 50 mm diameter (P/50) aluminum cylinder, 3 mm/s crosshead speed, and a load cell of 5 kg were used for all the samples. Data were collected and assessed using Exponent Connect 8.1.11.0 Lite software (Stable Micro Systems, Godalming, UK). At least three samples were tested for each material system.

### 4.6. Advanced Characterization of the Scaffolds

Small-angle X-ray scattering (SAXS) and wide-angle X-ray scattering (WAXS) measurements were conducted on the SWING beamline at the SOLEIL synchrotron. The wavelength of the X-ray was 1.0332 Å. The sample-to-detector distances were 1404.1 mm and 6504.1 mm for SAXS and 73.66 mm for WAXS, covering q-ranges from 0.00098 Å^−1^ to 0.67 Å^−1^ for SAXS and from 1.2 Å^−1^ to 4.4 Å^−1^ for WAXS. Data was accumulated for 2.0 s (at long distance) and 0.5 s (at short distance) at each spot for a total of 9 different spots to avoid beam damage and to verify uniformity. 2D data were radially integrated into 1D plots for data analysis.

Small-angle neutron scattering (SANS) measurements were carried out on the D11 instrument at ILL to study time-resolved changes while exposing samples to a humid environment. Cassettes with Kapton windows were used to hold the samples, and a gas-flow humidity generator was equipped to condition the samples in D_2_O vapor at a starting relative humidity (RH) of 2%, followed by an increase to over 90% for 16 h. Three configurations of wavelength, sample-to-detector distance, and measurement time were applied: (i) 4.6 Å, 1.6 m, 3 min; (ii) 4.6 Å, 12.7 m, 6 min; and (iii) 13.3 Å, 38.9 m, 6 min. The acquired data covered a q-range of 0.00046 Å^−1^ to 0.75 Å^−1^. 2D data were radially integrated into 1D plots for data analysis.

### 4.7. Cytocompatibility Assessment of the Scaffolds

Direct and indirect cytocompatibility studies were conducted according to the ISO 10993-5:2009 [33] standard guideline. Murine fibroblast line 3T3-J2 was selected as a standardized model for initial toxicity screening due to its high sensitivity to cytotoxic agents and its established use in evaluating the biological safety of medical devices. The murine fibroblast line 3T3-J2 was kindly provided by the Stem Cells and Aging group at Biogipuzkoa.

On the one hand, in order to evaluate the cytotoxicity produced by the extracts emitted by the scaffolds, 5000 cells were cultured in a 96-well cell culture plate in complete DMEM, supplemented with 10% (*v*/*v*) inactive FBS, 1% (*v*/*v*) P/S, and 1% (*v*/*v*) L-glutamine at 37 °C in a humidified incubator with a 5% CO_2_ atmosphere. At the same time, the scaffolds were kept for 24 h at 37 °C in a complete DMEM under constant stirring to facilitate the release of the extracts. Subsequently, the culture medium was replaced by scaffold extracts, leaving them for another 24 h.

On the other hand, the cytotoxicity by direct contact of the scaffold on the cells was evaluated. To accomplish this, 35,000 cells were seeded in a 24-well plate and maintained at 37 °C under an atmosphere of 5% CO_2_ and complete DMEM. Thereafter, a hydrated scaffold was placed on top of fibroblast-containing wells, and they were cultured for another 24 h. Finally, in both experiments, after the incubation period, the metabolic activity of the viable fibroblasts was evaluated by the CCK-8 kit according to the manufacturer’s instructions. After 2 h of incubation at 37 °C and 5% CO_2_, the absorbance was measured at 450 nm in the Halo Led 96 (Precisa Gravimetrics, Switzerland). The metabolic activity of cells without contact with extracts or scaffolds was considered 100% activity.

### 4.8. Long-Term Biocompatibility

#### 4.8.1. Cell Isolation

Briefly, adult pig ear cartilage was obtained post-mortem after removing the skin and perichondrium of the tissue. After slicing all tissue samples into small pieces, they were washed with washing medium composed of Hank’s balanced salt solution supplemented with 2% P/S and 2% Fungizone™. Afterwards, the fragments were digested in a digestion medium composed of DMEM containing 1.5 mg/mL of collagenase type II, 2% P/S, and 2% Fungizone™, and incubated overnight at 37 °C with gentle shaking. Subsequently, the digestion medium with the cells was filtered through a 70 μm nylon cell strainer in order to get rid of tissue rests and clots. After that, cells from the digested tissue were centrifuged at 462× *g* for 5 min and washed three times with washing medium. The cells were then resuspended in expansion medium consisting of low-glucose DMEM supplemented with 10% FBS and 1% P/S.

For the extraction of pig adipogenic mesenchymal stem cells (aMSCs), the entire surface of the skin was disinfected with povidone and 70% ethanol after removing the leg hair. Subsequently, an incision was made, excising a rectangular tissue section using a scalpel. Afterwards, the remains of venous tissue or capillaries that could be present were cleaned with the help of tweezers, and the fatty tissue under the skin was cut, scraping it very carefully. Adipogenic tissue was sliced into small pieces and transferred into a collection tube, where it was washed with washing medium overnight at 4 °C. The next step was the incubation of the tissue with collagenase type II (0.2% *w*/*v*) in washing medium at 37 °C for 1 h at a constant and vigorous agitation. Afterwards, the digested tissue was centrifuged for 7 min at 300× *g*. The generated pellet was then strained with a 100 µm cell strainer, resuspended in washing medium, incubated for 5 min, and centrifuged twice before seeding in a culture flask.

Viable cells were determined using the trypan blue dye exclusion technique. Isolated chondrocytes and aMSCs were then cultured until confluence at 37 °C in a humidified CO_2_ incubator, replacing the medium every 3 days.

#### 4.8.2. In Vitro 3D Scaffold Culture

A total amount of 50,000 chondrocytes or aMSCs was seeded on top of the scaffolds, allowing them to adhere and grow for 5 days in culture medium in a humidified CO_2_ incubator, changing the medium twice a week. Then, the scaffolds were cultured under hypoxic conditions (1% O_2_) with pro-chondrogenic medium (high-glucose DMEM, 5% FBS, 1X ITS, 100 nM dexamethasone, 100 μg/mL ascorbic acid, 1% P/S, and glutamine) for 4 weeks. Samples of both cellular types growing on top of scaffolds were taken at 7, 14, 21, and 28 days.

#### 4.8.3. Long-Term Viability Assessment

The viability of seeded cells (chondrocytes and aMSCs) was qualitatively assessed using a Live/Dead kit. Thus, cultured scaffolds collected at specific time points (7, 14, 21, and 28 days) were subsequently washed three times with PBS for 5 min. Afterwards, a staining solution of ethidium homodimer and Calcein-AM diluted in PBS was added to those scaffolds. After 15 min, images were taken with a Zeiss LSM 900 confocal microscope (Leica, Madrid, Spain). On the other hand, the metabolic activity of the cells was quantitatively assessed over the assay time (7, 14, 21, and 28 days) using the CCK-8 kit (Sigma Aldrich, Madrid, Spain).

#### 4.8.4. Biochemical Evaluation of Neo-Cartilage Formation

The determination of sulfated glycosaminoglycan (sGAG) deposition as an indicator of the extracellular matrix deposited by the cells was carried out using the Blyscan Kit (Sigma Aldrich, Madrid, Spain) according to the manufacturer’s instructions. The absorbance of each sample was read at 656 nm in a HALO LED 96 microplate reader (Precisa Gravimetrics, Dietikon Switzerland).

### 4.9. Statistical Analysis

The SPSS Statistics 25.0 software program (IBM, Armonk, USA) was used to perform the statistical analysis. All results are presented as the mean ± standard deviation. The distribution of the data was assessed by the Shapiro–Wilk test. For normally distributed data, Student’s *t*-test or one-way ANOVA was applied for differences between two groups or multiple comparisons, respectively. The Tukey post hoc test was applied for multiple comparisons. For non-normally distributed data, the Mann–Whitney U test, nonparametric analysis, or the Kruskal–Wallis test with Dunn’s multiple comparisons tests were applied. In all cases, *p* values ≤ 0.05 were considered significantly different.

## Figures and Tables

**Table 1:** Carreau–Yasuda rheological model parameters for 0CHI, 20CHI, and 40CHI samples.

Sample	$\eta_{0}$ (Pa·s)	$\eta_{\infty }$ (Pa·s)	λ (s)	*a*	*n*	R^2^
0CHI	4030.61	0.32	20.72	7.37	0.20	0.99
20CHI	952.07	0.14	30.64	1.41	0.21	1.00
40CHI	189.66	0.17	34.27	5.33	0.29	1.00

**Table 2:** Hydrolytic degradation, compressive modulus, and cohesiveness of 0CHI, 20CHI, and 40CHI samples. Means with different letters in the same column are significantly different (*p* ≤ 0.05). Statistics were determined using one-way ANOVA followed by Tukey’s post hoc test (n = 5).

Sample	Degradation (%)	Compressive Modulus (kPa)	Cohesiveness
0CHI	24.9 ± 0.2 ^a^	4.8 ± 0.3 ^a^	0.94 ± 0.02 ^a^
20CHI	25.0 ± 0.8 ^a^	12.8 ± 0.6 ^b^	0.97 ± 0.01 ^b^
40CHI	25.8 ± 0.5 ^a^	8.6 ± 0.7 ^c^	0.97 ± 0.01 ^b^

## Data Availability

Data will be made available on request.

## References

[1] Klarmann G.J., Piroli M.E., Loverde J.R., Nelson A.F., Li Z., Gilchrist K.H., Gaston J.D., Ho V.B. (2023). 3D Printing a Universal Knee Meniscus Using a Custom Collagen Ink. Bioprinting.

[2] Dewle A., Pathak N., Rakshasmare P., Srivastava A. (2020). Multifarious Fabrication Approaches of Producing Aligned Collagen Scaffolds for Tissue Engineering Applications. ACS Biomater. Sci. Eng..

[3] Chen Q., Pei Y., Tang K., Albu-Kaya M.G. (2023). Structure, Extraction, Processing, and Applications of Collagen as an Ideal Component for Biomaterials—A Review. Collagen Leather.

[4] Guo C., Wu J., Zeng Y., Li H. (2023). Construction of 3D Bioprinting of HAP/Collagen Scaffold in Gelation Bath for Bone Tissue Engineering. Regen. Biomater..

[5] Houška M., Landfeld A., Novotná P., Strohalm J., Šupová M., Suchý T., Chlup H., Skočilas J., Štípek J., Žaloudková M. (2023). Properties of Bovine Collagen as Influenced by High-Pressure Processing. Polymers.

[6] Andonegi M., Heras K.L., Santos-Vizcaíno E., Igartua M., Hernandez R.M., de la Caba K., Guerrero P. (2020). Structure-Properties Relationship of Chitosan/Collagen Films with Potential for Biomedical Applications. Carbohydr. Polym..

[7] Adhikari J., Dasgupta S., Das P., Gouripriya D.A., Barui A., Basak P., Ghosh M., Saha P. (2024). Bilayer Regenerated Cellulose/Quaternized Chitosan-Hyaluronic Acid/Collagen Electrospun Scaffold for Potential Wound Healing Applications. Int. J. Biol. Macromol..

[8] Elizalde-Cárdenas A., Ribas-Aparicio R.M., Rodríguez-Martínez A., Leyva-Gómez G., Ríos-Castañeda C., González-Torres M. (2024). Advances in Chitosan and Chitosan Derivatives for Biomedical Applications in Tissue Engineering: An Updated Review. Int. J. Biol. Macromol..

[9] Zhang X., Cheng F., Islam M.R., Li H. (2024). The Fabrication of the Chitosan-Based Bioink for *in Vitro* Tissue Repair and Regeneration: A Review. Int. J. Biol. Macromol..

[10] Mohammadpour Z., Kharaziha M., Zarrabi A. (2023). 3D-Printing of Silk Nanofibrils Reinforced Alginate for Soft Tissue Engineering. Pharmaceutics.

[11] Agarwal T., Chiesa I., Costantini M., Lopamarda A., Tirelli M.C., Borra O.P., Varshapally S.V.S., Kumar Y.A.V., Koteswara Reddy G., De Maria C. (2023). Chitosan and Its Derivatives in 3D/4D (Bio) Printing for Tissue Engineering and Drug Delivery Applications. Int. J. Biol. Macromol..

[12] Tajik S., Garcia C.N., Gillooley S., Tayebi L. (2023). 3D Printing of Hybrid-Hydrogel Materials for Tissue Engineering: A Critical Review. Regen. Eng. Transl. Med..

[13] Schwab A., Levato R., D’Este M., Piluso S., Eglin D., Malda J. (2020). Printability and Shape Fidelity of Bioinks in 3D Bioprinting. Chem. Rev..

[14] Lan X., Adesida A., Boluk Y. (2022). Rheological and Viscoelastic Properties of Collagens and Their Role in Bioprinting by Micro-Extrusion. Biomed. Mater..

[15] Dick A., Bhandari B., Dong X., Prakash S. (2020). Feasibility Study of Hydrocolloid Incorporated 3D Printed Pork as Dysphagia Food. Food Hydrocoll..

[16] Bercea M. (2023). Rheology as a Tool for Fine-Tuning the Properties of Printable Bioinspired Gels. Molecules.

[17] Zheng T., Tang P., Shen L., Bu H., Li G. (2021). Rheological Behavior of Collagen/Chitosan Blended Solutions. J. Appl. Polym. Sci..

[18] Gholap A.D., Rojekar S., Kapare H.S., Vishwakarma N., Raikwar S., Garkal A., Mehta T.A., Jadhav H., Prajapati M.K., Annapure U. (2024). Chitosan Scaffolds: Expanding Horizons in Biomedical Applications. Carbohydr. Polym..

[19] Marin M.M., Ianchis R., Leu Alexa R., Gifu I.C., Kaya M.G.A., Savu D.I., Popescu R.C., Alexandrescu E., Ninciuleanu C.M., Preda S. (2022). Development of New Collagen/Clay Composite Biomaterials. Int. J. Mol. Sci..

[20] Maxwell C.A., Wess T.J., Kennedy C.J. (2006). X-Ray Diffraction Study into the Effects of Liming on the Structure of Collagen. Biomacromolecules.

[21] Andonegi M., Correia D.M., Costa C.M., Lanceros-Mendez S., de la Caba K., Guerrero P. (2022). Tailoring Physicochemical Properties of Collagen-Based Composites with Ionic Liquids and Wool for Advanced Applications. Polymer.

[22] Bak S.Y., Lee S.W., Choi C.H., Kim H.W. (2018). Assessment of the Influence of Acetic Acid Residue on Type I Collagen during Isolation and Characterization. Materials.

[23] Durga R., Jimenez N., Ramanathan S., Suraneni P., Pestle W.J. (2022). Use of Thermogravimetric Analysis to Estimate Collagen and Hydroxyapatite Contents in Archaeological Bone. J. Archaeol. Sci..

[24] Kasimova M.R., Velázquez-Campoy A., Nielsen H.M. (2011). On the Temperature Dependence of Complex Formation between Chitosan and Proteins. Biomacromolecules.

[25] Liao W., Guanghua X., Li Y., Shen X.R., Li C. (2018). Comparison of Characteristics and Fibril-Forming Ability of Skin Collagen from Barramundi (*Lates calcarifer*) and Tilapia (*Oreochromis niloticus*). Int. J. Biol. Macromol..

[26] Torre M., Giannitelli S.M., Mauri E., Gori M., Bucciarelli A., Mozetic P., Gigli G., Trombetta M., Rainer A. (2023). Printability Assessment Workflow of a Thermosensitive Photocurable Biomaterial Ink for Microextrusion Bioprinting. Bioprinting.

[27] Swanson W.B., Omi M., Zhang Z., Nam H.K., Jung Y., Wang G., Ma P.X., Hatch N.E., Mishina Y. (2021). Macropore Design of Tissue Engineering Scaffolds Regulates Mesenchymal Stem Cell Differentiation Fate. Biomaterials.

[28] Eckstein F., Tieschky M., Faber S., Englmeier K.-H., Reiser M. (1999). Functional Analysis of Articular Cartilage Deformation, Recovery, and Fluid Flow Following Dynamic Exercise in Vivo. Anat. Embryol..

[29] Krishani M., Shin W.Y., Suhaimi H., Sambudi N.S. (2023). Development of Scaffolds from Bio-Based Natural Materials for Tissue Regeneration Applications: A Review. Gels.

[30] (2025). Biological Evaluation of Medical Devices—Part 1: Evaluation and Testing Within a Risk Management Process. https://www.iso.org/standard/10993-1.

[31] Zhao M., Gao X., Wei J., Tu C., Zheng H., Jing K., Chu J., Ye W., Groth T. (2022). Chondrogenic Differentiation of Mesenchymal Stem Cells through Cartilage Matrix-Inspired Surface Coatings. Front. Bioeng. Biotechnol..

[32] Schneider C.A., Rasband W.S., Eliceiri K.W. (2012). NIH Image to ImageJ: 25 Years of Image Analysis. Nat. Methods.

[33] *ISO 10993-5:2009*; Biological Evaluation of Medical Devices Part 5: Tests for In Vitro Cytotoxicity. https://www.iso.org/standard/36406.html.

